# Author Correction: Development and clinical validation of Swaasa AI platform for screening and prioritization of pulmonary TB

**DOI:** 10.1038/s41598-023-37429-x

**Published:** 2023-06-26

**Authors:** Gayatri Devi Yellapu, Gowrisree Rudraraju, Narayana Rao Sripada, Baswaraj Mamidgi, Charan Jalukuru, Priyanka Firmal, Venkat Yechuri, Sowmya Varanasi, Venkata Sudhakar Peddireddi, Devi Madhavi Bhimarasetty, Sidharth Kanisetti, Niranjan Joshi, Prasant Mohapatra, Kiran Pamarthi

**Affiliations:** 1 Andhra Medical College, Visakhapatnam, India; 2 Salcit Technologies, Jayabheri Silicon Towers, Hyderabad, India; 3 Centre for Cellular and Molecular Platforms, Bengaluru, India; 4 Department of Computer Science, University of California, Davis, USA

Correction to: *Scientific Reports* 10.1038/s41598-023-31772-9, published online 23 March 2023

The original version of this Article contained errors in Table 6, where the “FP” and “FN” values were interchanged. The correct and incorrect values appear below.

Incorrect:

**Table:** 

	TB likely - Yes	TB likely - No
TB - Yes	15 (TP)	5 (FN)
TB - No	9 (FP)	36 (TN)

Correct:

**Table:** 

	TB likely - Yes	TB likely - No
TB - Yes	15 (TP)	9 (FN)
TB - No	5 (FP)	36 (TN)

The original Article has been corrected.

